# Fuzzy inference-based LSTM for long-term time series prediction

**DOI:** 10.1038/s41598-023-47812-3

**Published:** 2023-11-21

**Authors:** Weina Wang, Jiapeng Shao, Huxidan Jumahong

**Affiliations:** 1 College of Information and Control Engineering, Jilin Institute of Chemical Technology, Jilin, 132022 China; 2 School of Network Security and Information technology, YiLi Normal University, Yining, 835000 China

**Keywords:** Environmental sciences, Energy science and technology, Mathematics and computing

## Abstract

Long short-term memory (LSTM) based time series forecasting methods suffer from multiple limitations, such as accumulated error, diminishing temporal correlation, and lacking interpretability, which compromises the prediction performance. To overcome these shortcomings, a fuzzy inference-based LSTM with the embedding of a fuzzy system is proposed to enhance the accuracy and interpretability of LSTM for long-term time series prediction. Firstly, a fast and complete fuzzy rule construction method based on Wang–Mendel (WM) is proposed, which can enhance the computational efficiency and completeness of the WM model by fuzzy rules simplification and complement strategies. Then, the fuzzy prediction model is constructed to capture the fuzzy logic in data. Finally, the fuzzy inference-based LSTM is proposed by integrating the fuzzy prediction fusion, the strengthening memory layer, and the parameter segmentation sharing strategy into the LSTM network. Fuzzy prediction fusion increases the network reasoning capability and interpretability, the strengthening memory layer strengthens the long-term memory and alleviates the gradient dispersion problem, and the parameter segmentation sharing strategy balances processing efficiency and architecture discrimination. Experiments on publicly available time series demonstrate that the proposed method can achieve better performance than existing models for long-term time series prediction.

## Introduction

Time series forecasting (TSF) is the process of analyzing time series data using statistics and modeling to make predictions and inform straegic decision-making. TSF plays a vital role in various domains, especially in the fields of financial management^1, 2^, social network^3–5^, medical science^6–8^, and industrial engineering^9, 10^. Therefore, there is a growing consensus that it is of great importance to enhance the accuracy and interpretability of TSF due to its widespread application.

The conventional techniques for time series data analysis have assumed a linear relationship between past and future values for the purpose of prediction. This kind of model is represented by the linear regression approach, such as ARIMA-based models^11^, and has shown good results for short-term predictions. However, their performance deteriorates significantly when the parameters are not selected properly, and the arima based model is not suitable for time series data with weak periodic characteristics^6, 8^. Chen et al.^5^ propose a dynamic linear model to extract the systematical time-series dynamic and volatility features to achieve more accurate prediction, and achieved some success. Today, deep learning-based models have generated great success, especially in long-term time series prediction tasks, where deep learning models significantly outperform conventional linear models. Artificial neural network (ANN) has been a powerful tool for TSF by virtue of its universal approximation capability, nonlinear modeling capability^12, 13^. Recurrent neural network (RNN)^14^ can use internal memory cells to handle temporal data, and is employed to model history and future states^15–17^. However, the gradient disappearance problem of the RNN makes its performance limited. To overcome this problem, long short-term memory (LSTM) constructs the “gate” to determine the remembering and forgetting information to obtain the long-term memory of historical data. Ma et al.^18^ combine LSTM and bidirectional LSTM networks to perform transportation prediction. Bandara et al.^19^ propose a decomposition-based unified network architecture (LSTM-MSNet) to predict multiple seasonal time series. LSTM-based models, however, are notorious for their limited ability to effectively utilize the latest data and accurately model long sequences and cycles in time series. Additionally, the performance of LSTM for long-term prediction is hindered by the amplification of small errors inherent in the model. Transformer-based time series model is currently a popular research direction, and its modeling ability is incomparable to traditional neural networks. Its inherent advantages in processing and predicting long sequence data make it perform excellently in most temporal tasks^20, 21^. However, its O($n^2$) algorithm complexity leads to an explosive increase in memory usage when executing long sequence data. Therefore, current research on it mostly focuses on optimizing algorithm efficiency and reducing algorithm complexity^22–24^.

Fuzzy system characterized by universal approximation capability and outstanding interpretability, providing an effective paradigm for handling uncertain data, representing latent knowledge, and exhibiting the inference process^25^. Some models attempt to enhance deep learning-based models by embedding fuzzy set theory, such as fuzzy deep convolutional neural network^26^, deep fuzzy echo state network^27^, and fuzzy recurrent neural network^28^, etc. Especially for time series forecasting, the related works have been proposed to integrate the fuzzy system with LSTM carrying the advantages of both fuzzy logic and deep learning. Li et al.^29^ propose a Type-2 fuzzy LSTM neural network to perform traffic volume prediction. Tang et al.^30^ propose a granule time series forecasting model by integrating the trend fuzzy granule and LSTM network. The innovations of these models are mainly reflected in taking the fuzzy information as input data and training the network’s parameters with a fuzzy system. However, the overall structure of LSTM has not changed, and the interpretability of the fuzzy system has been shrunken to some extent.

The extraction of fuzzy rules from the training data is a crucial component in modeling a fuzzy system. The Wang–Mendel (WM) model is a powerful tool for directly extracting fuzzy rules using only one pass of the training data^31, 32^. The effectiveness of the WM model could be greatly degraded due to the excessive generation of fuzzy rules. To overcome this issue, an improved WM model utilizing fuzzy c-means is proposed^33^. But, there is still a challenging task to determine the number of clustering. Zhai et al.^34^ propose an on-line WM fuzzy inference model, which can adaptively acquire the fuzzy rules from training data. However, the performance of the model could be limited due to the redundant rules and the lacking rules not covered by the training data.

Taking all the above observations into consideration, this paper proposes a fuzzy inference-based LSTM for time series forecasting, which enhance the accuracy and interpretability of LSTM with by embedding fuzzy system. To improve the computational efficiency and completeness of WM model, a fuzzy rule base construction method based on WM model is proposed. Then, the fuzzy prediction model based on the improved WM model is constructed. Finally, the fuzzy inference-based LSTM is proposed to carry out prediction by integrating the fuzzy prediction fusion, the strengthening memory layer, and the parameter segmentation sharing strategy into the LSTM network. In summary, the main contributions of this model are as follows:

(i) A fast and complete fuzzy rule construction method based on the WM model is proposed, which can enhance the computational efficiency and completeness of the WM model by fuzzy rules simplification and complement strategies.
(ii) Strengthening memory layer is constructed by integrating the current output with the cell state, which can strengthen the long-term memory and alleviate the gradient dispersion problem of LSTM.
(iii) Parameter segmentation sharing strategy by dividing the overall output layer into different parts is proposed, which can balance processing efficiency and architecture discrimination.
(iv) Fuzzy inference-based LSTM with the embedding of a fuzzy system is proposed, which can enhance the accuracy and interpretability of LSTM for long-term time series prediction.
(v) Extensive experiments demonstrate the better performance of the proposed method in comparison with related models.

## Prerequisites

This paper focuses on improving the interpretability and accuracy of deep neural network based on the fuzzy inference model in tackling the time series prediction problem. This section mainly introduces two related methods, LSTM and WM model.

### Long Short-Term Memory Neural Network (LSTM)

RNN has achieved good performance in processing and learning time series information, but it cannot successfully learn long-term dependencies due to gradient explosion or gradient disappearance problems. LSTM is an extension for RNNs, which introduces the “gate” cell to retain and learn long-term dependencies. LSTM network can capture important features from inputs and store the information over a long period of time, thus it has achieved good results in long-term forecasting. In general, the critical components of LSTM network architecture consists of three gates: forget, input, and output gates denoted by *f*, *i* and *o*, respectively. The detailed description of the calculation procedure for each gate is shown as follows:

**(1) Forget Gate.** Determine what information needs to be retained in the memory cell with the help of sigmoid function. The output is expressed as follows:

1 $$\begin{aligned} f_t = \sigma (W_{fx}\cdot x_t+W_{fh} \cdot h_{t-1}+b_f) \end{aligned}$$

where $x_t$ and $h_{t-1}$ represent input and hidden state at time step *t* and $t-1$, respectively. *W* represents weight matrices, $b_f$ represents a constant bias, and $\sigma (\cdot )$ represents sigmoid function.

**(2) Input Gate.** Determine whether the new information should be saved to the memory cell by the sigmoid layer and tanh layer. The outputs of the two layers are computed in the following form:

2 $$\begin{aligned} i_t = \sigma (W_{ix}\cdot x_t+W_{ih} \cdot h_{t-1}+b_i) \end{aligned}$$

3 $$\begin{aligned} \tilde{c}_t = \textrm{tanh}(W_{cx}\cdot x_t+W_{ch} \cdot h_{t-1}+b_c) \end{aligned}$$

The update of the memory cell is achieved by the combination of these two layers, where the current memory is obtained by retaining previous information and introducing new cell state information. The mathematical equation is expressed in the following form:

4 $$\begin{aligned} c_t=f_t \circ c_{t-1}+i_t \circ \tilde{c}_t \end{aligned}$$

where $c_{t}$ represents cell state at time step *t*, $\circ$ denotes the Hadamard product.

**3) Output Gate.** Determine what part of the memory contributes to the current put and map the output between $-1$ and 1 by tanh function. The outputs can be computed by the following equations:

5 $$\begin{aligned} o_t = \sigma (W_{ox}\cdot x_t+W_{oh} \cdot h_{t-1}+b_o) \end{aligned}$$

6 $$\begin{aligned} h_t=o_t \cdot \textrm{tanh}(c_t) \end{aligned}$$

### Wang–Mendel model

WM model is a simple and powerful tool for generating the fuzzy rule base from sample data. However, the effectiveness of the WM model could be greatly degraded due to the huge amount of data. Each training data generates a fuzzy rule resulting that the rule extraction strategy is not efficient enough. Thus, improving the rule generation mechanism becomes crucial, and a fast and complete fuzzy rule base construction method based on the WM model will be proposed. The simplification strategy for redundant rules and conflict rules is proposed to simplify the fuzzy rule base. The complement strategy is proposed to complement the fuzzy rule base.

**Fuzzy rules extraction.** Given the time series, define the length of the antecedents and consequents of the fuzzy rule, and divide several fuzzy subsets of each variable of the antecedents to extract input-output sample pairs. Each feature of the input-output sample pair can be assigned to the fuzzy set with the highest membership degree, and these membership degrees are used to calculate the weight of the fuzzy rule, and finally an unorganized fuzzy rule base is generated.

**Fuzzy rule arrangement.** When the sample size is large, it is easy to generate redundant rules. To solve the above problem, when adding a rule to the rule base, first check whether the antecedents of the rule already exist in the rule base. If it does not exist, add it to the rule base; Otherwise, save the rule with the highest weight.

**Fuzzy rule-based prediction.** A central antifuzzy inference machine is used to organize rules with the same antecedents in the fuzzy rule base, obtain the consequent of the rules for fuzzy inference, and get the final fuzzy rule inference base. .

The drawback of this model is that the generated fuzzy rule library lacks good completeness and robustness, resulting in low model accuracy. Therefore, in order to improve the accuracy of the model, we need to optimize the method of constructing a fuzzy rule inference system to quickly and comprehensively construct a fuzzy rule inference system.

## The proposed fuzzy prediction model

**Figure 1:**
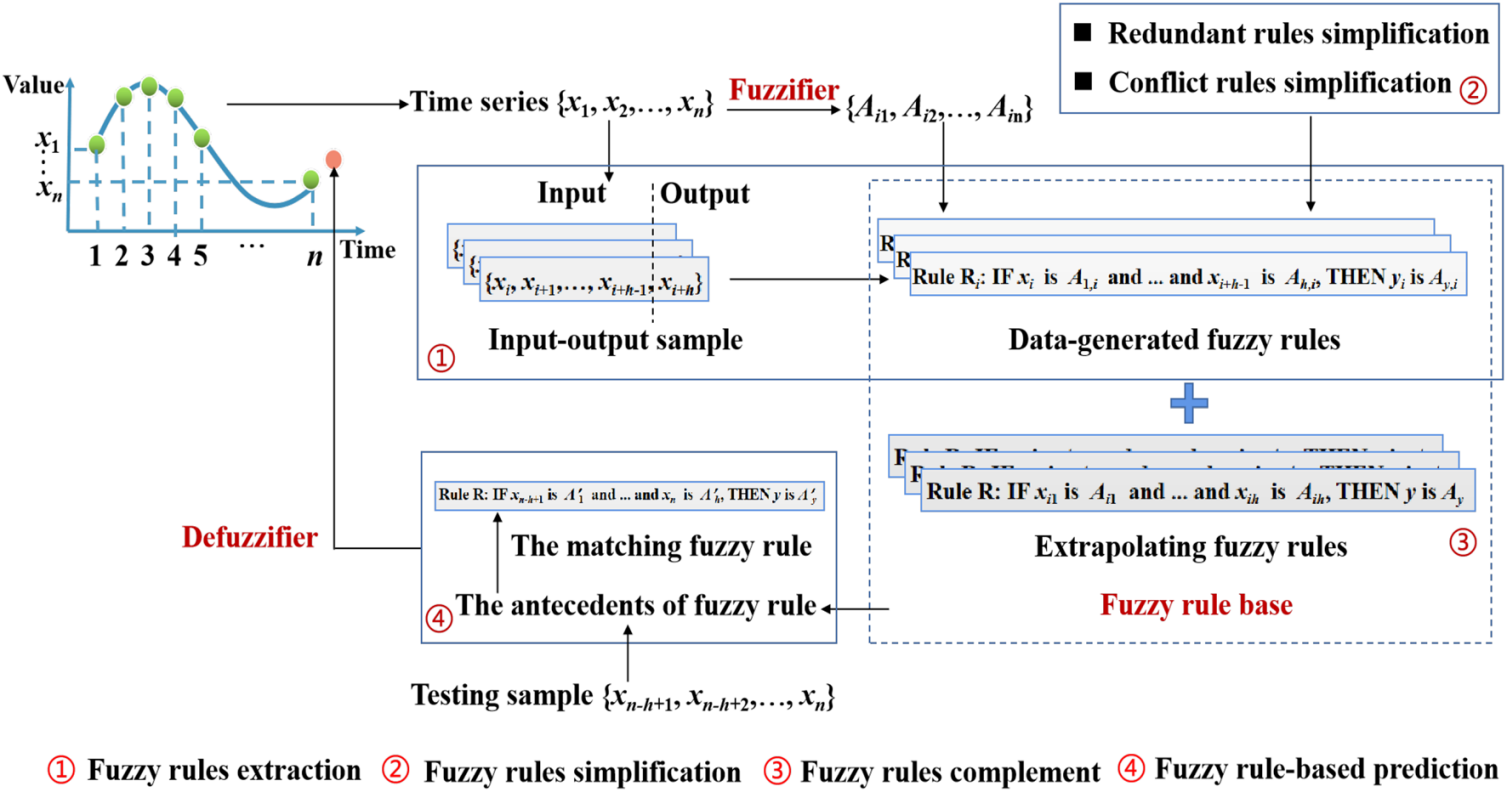
Framework of the proposed fuzzy prediction model.

The construction of fuzzy rule base is crucial for fuzzy rule-based prediction model. The fuzzy rule base constructed based on WM model may be have redundant rules, and lack correspondence rules for new available sample due to the fuzzy regions uncovered by training data. To improve the computational efficiency and completeness of WM model, a fast and complete fuzzy rule base construction method based on WM model is proposed, then the prediction is performed based on the fuzzy rule base. The framework of the proposed fuzzy prediction model is shown in Fig. 1. In what follows, we explain the detailed steps of the proposed model.

### Fuzzy rules extraction

**Figure 2:**
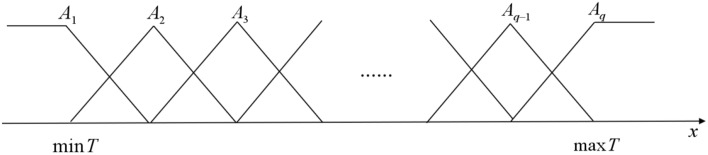
Triangular fuzzy sets.

Given the time series $T=\{x_{1},x_{2},\ldots , x_{n}\}$, each input-output sample pair for training can be constructed as $\{x_{i},x_{i+1},\ldots , x_{i+h-1}, y_{i}\}$, $i=1, 2, \ldots , n-h$, where $\{x_{i},x_{i+1}, \ldots , x_{i+h-1}\}$ is input sample, *h* is the length of input sample, and $y_{i}=x_{i+h}$ is output sample. The domain of discourse is divided into *q* regions, then define the triangular fuzzy sets $A_{1}, A_{2},\ldots , A_{q}$ based on these regions shown in Fig. 2.

Each feature of input-output sample pair can be assigned to the fuzzy set defined with the highest membership degree, i.e. $x_{i}$ is fuzzified into $A_{1,i}$ with the membership degree $U_{1,i}$. The fuzzy rules can be extraction using WM method as follows:

7 $$\begin{aligned} {{\textbf {Rule}}}\ R_i: {{\textbf {IF}}}\ x_{i}\ is \ A_{1,i} \ \textrm{and} \ \cdots \ \textrm{and} \ x_{i+h-1} \ is \ A_{h,i}, {{\textbf {THEN}}} \ y_i \ is \ A_{y,i} \end{aligned}$$

where $A_{j,i}$ is the *j*th antecedent, and $A_{y,i}$ is the consequence. The rule that is generated from the training data be called data-generated rules, and fuzzy rule base can be constructed and denoted as $R=\{R_1, R_2, \ldots , R_{n}\}$.

### Fuzzy rules simplification

When the size of sample set is massive, a large number of fuzzy rules are generated. There are many fuzzy rules with same characteristics, such as redundant rules and conflict rules. Redundant rules refer to those rules with the same antecedents and consequence, and conflict rules are the rules that have the same antecedent but different consequences. To simplify the fuzzy rule base, the simplification strategy for redundant rules and conflict rules is proposed as follows:

**(1) Redundant rules simplification.** Find the group of date-generated rules that have the same antecedents and consequences, and then keep only one fuzzy rule among them and delete the group from the fuzzy rule base.

**(2) Conflict rules simplification.** Find the group of date-generated rules that have the same antecedents but different consequences, and then integrate the information of all fuzzy rules in the group to generate a new fuzzy rule. Delete the group from the fuzzy rule base and add the new fuzzy rule to the fuzzy rule base.

The process of conflict rules simplification are explained as follows. Assume the group found are $R_{1}^{\prime }, R_{2}^{\prime }, \ldots , R_{m}^{\prime }$, the fuzzy rule $R_i^{\prime }$ can be expressed as:

$$\begin{aligned} {{\textbf {Rule}}}\ R_i^{\prime }: {{\textbf {IF}}}\ x_{i1}\ \textrm{is} \ A_{i1} \ \textrm{and} \ \cdots \ \textrm{and} \ x_{ih} \ \textrm{is} \ A_{ih}, {{\textbf {THEN}}} \ y_i \ \textrm{is} \ A_{y_{i}} \end{aligned}$$

The weight of each fuzzy rule $R_i^{\prime }$ can be computed by the product of membership function values for each antecedent:

8 $$\begin{aligned} W_i= \Pi _{j=1}^{h} U_{j,i} \end{aligned}$$

where $U_{j,i}$ is the membership degree of $x_{ij}$ to $A_{ij}$. Then the value can be obtained by using the center-average defuzzification mechanism:

9 $$\begin{aligned} \hat{y}=\frac{\sum _{i=1}^{m} W_i\cdot \bar{y}_{i}}{\sum _{i=1}^{m}W_i} \end{aligned}$$

where $\bar{y}_{i}$ is the central value of fuzzy set $A_{y_i}$. Assuming that $A_{\hat{y}}$ is the fuzzy set on which $\hat{y}$ achieves the maximum membership, the new fuzzy rule is generated as follows:

10 $$\begin{aligned} {{\textbf {Rule}}}\ R: {{\textbf {IF}}}\ x_{i1}\ {\textrm{is}} \ A_{i1} \ {\textrm{and}} \ \cdots \ {\textrm{and}} \ x_{ih} \ {\textrm{is}} \ A_{ih}, {{\textbf {THEN}}} \, {{\hat{y}}_{i}} \, {\text{is}} \, {{A}_{\hat{y}}} \end{aligned}$$

### Fuzzy rules complement

The fuzzy rules are extracted from the fuzzy regions that contain sample data, thus the data-generated fuzzy rule base is in general not complete. To extrapolate the data-generated fuzzy rule base over the regions not covered by these obtained rules, the fuzzy rule base should be complemented to cover the whole domain of discourse. Especially for the forecasting problem, a complete fuzzy rule base is crucial because the rules should be well-defined at all samples in the domain of discourse. To complement the fuzzy rule base, the complement strategy is proposed as the following three steps.

**Step 1)** For each combination of antecedents that does not appear in the fuzzy rule base, find the group of data-generated fuzzy rules that differ from the combination in only *i* antecedents, and call this group the *i*-group. Determine the first group that is not an empty, i.e. *t*-group.

**Step 2)** For all fuzzy rules in *t*-group, compute:

11 $$\begin{aligned} \hat{y}=\frac{\sum _{i=1}^{n_{t}} \bar{y}^{i}}{n_{t}} \end{aligned}$$

where $n_{t}$ is the number of fuzzy rules in *t*-group, $y^{i}$ is the central value of fuzzy set that is the consequence of *i*th fuzzy rule in *t*-group.

**Step 3)** Find the fuzzy set $A_{\hat{y}}$ on which $\hat{y}$ achieves the maximum membership. Assuming that the combination of antecedents is “$x_{i1}\ \textrm{is} \ A_{i1} \ \textrm{and} \ \cdots \ \textrm{and}$ $\ x_{ih} \ \textrm{is} \ A_{ih}$”, the extrapolating rule is generated as:

12 $$\begin{aligned} {{\textbf {Rule}}}\ R: {{\textbf {IF}}}\ x_{i1}\ {\textrm{is}} \ A_{i1} \ {\textrm{and}} \ \cdots \ {\textrm{and}} \ x_{ih} \ {\textrm{is}} \ {A}_{ih}, {{\textbf {THEN}}} \, {\hat{y}} \, {\text{is}} \, {{A}_{\hat{y}}} \end{aligned}$$

The process is repeated until all the extrapolating rules are constructed. The complete fuzzy rule base can be obtained by integrating the extrapolating rules and data-generated rules.

### Fuzzy rule-based prediction

Let $\{x_{n-h+1}, x_{n-h+2}, \ldots , x_{n}\}$ be the testing sample, and each feature $x_{i}^{\prime }$ is fuzzified into a fuzzy set $A_{i}^{\prime }$. The antecedents of fuzzy rule is obtained as “$x_{n-h+1}\ \textrm{is} \ A_{1}^{\prime } \ \textrm{and} \ \cdots \ \textrm{and} \ x_{n} \ \textrm{is} \ A_{h}^{\prime }$”, and the matching fuzzy rule can be extracted from fuzzy rule base shown as:

13 $$\begin{aligned} {{\textbf {Rule}}}\ R: {{\textbf {IF}}}\ x_{n-h+1}\ \textrm{is} \ A_{1}^{\prime } \ \textrm{and} \ \cdots \ \textrm{and} \ x_{n} \ \textrm{is} \ A_{h}^{\prime }, {{\textbf {THEN}}} \ y \ \textrm{is} \ A_{y}^{\prime } \end{aligned}$$

Predicted value can be obtained by $\hat{y}=y^{\prime }$, where $y^{\prime }$ is the center of fuzzy set $A_{y}^{\prime }$.

In this section, an improved Wang–Mendel model for rapid construction of fuzzy inference systems is proposed, which improves the shortcomings of the incomplete fuzzy rule inference base that the Wang–Mendel model may generate by using a simpler way. Thus a complete fuzzy rule inference base is built. In the process of building this fuzzy inference system, there is not much extra time and computational overhead.

The addition of the fuzzy prediction module will affect the computational efficiency of the model. In the experiment, we improve the computational efficiency of the fuzzy prediction module as much as possible through the following methods.

1) The data in the input part of the experiment is fixed. To reduce the calculation cost of the fuzzy rule module, the construction of the fuzzy rule base is performed offline in advance.

2) For the data in the prediction part of the experiment, the branch bound search algorithm is used to reduce the computational cost when the fuzzy prediction inference base is used to find the corresponding rules.

## Fuzzy inference-based LSTM for time series prediction

**Figure 3:**
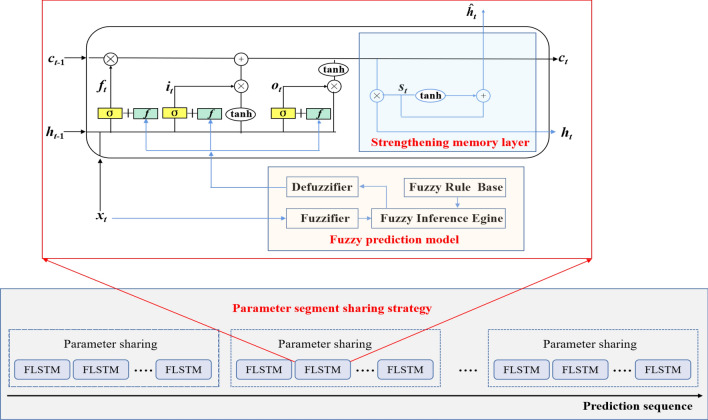
Framework of FLSTM model.

In this section, the fuzzy inference-based LSTM (FLSTM) for time series forecasting is proposed. The proposed method incorporates the fuzzy prediction fusion, the strengthening memory layer, and the parameter segment sharing strategy into the LSTM network. Fuzzy prediction fusion model combines the fuzzy prediction with the three gates in LSTM to enhance the fuzzy reasoning capacity of the network. Strengthening memory layer integrates the hidden state and the cell state to strengthen the long-term memory. Parameter segment sharing strategy divides the overall output layer into different parts to balance processing efficiency and architecture discrimination. The proposed forecasting model is shown in Fig. 3, and described in detail in the following section.

### Fuzzy prediction fusion

The fuzzy prediction model is embedded in the LSTM to enhance the network reasoning capability and interpretability. The fuzzy rule can capture the dynamic characteristic of data change, and the reasoning relationship between the latest information and the historical information is extracted in the form of rules. The fuzzy prediction model can take full advantage of the latest information to prediction future behavior. Therefore, the LSTM combines with fuzzy prediction model can effectively overcome the lacking in the utilization of latest data.

LSTM utilizes gate cell to control information flow in recurrent computations. Therefore, the input gate, forget gate, and output gate are combined with fuzzy prediction to produce new output, which can integrate the fuzzy prediction information into the recurrent computations. The mathematical expressions can be expressed as follows:

14 $$\begin{aligned} f^{(f)}_t = \sigma (W_{fx}\cdot x_t+W_{fh} \cdot h_{t-1}+W_{ff} \cdot r_{t}+b_f) \end{aligned}$$

15 $$\begin{aligned} i^{(f)}_t = \sigma (W_{ix}\cdot x_t+W_{ih} \cdot h_{t-1}+W_{if} \cdot r_{t}+b_i) \end{aligned}$$

16 $$\begin{aligned} o^{(f)}_t = \sigma (W_{ox}\cdot x_t+W_{oh} \cdot h_{t-1}+W_{of} \cdot r_{t}+b_o) \end{aligned}$$

where $r_{t}$ is output of fuzzy prediction model at time step *t*, and $W_{ff}, W_{if}, W_{of}$ are weight matrices of $r_{t}$ for input gate, forget gate, and output gate, respectively.

After the training of model, these weights can represent the strengths of the fuzzy rules in the different gates, thus the proposed input gate, forget gate, and output gate make the results more interpretable. Meanwhile, the fusion of fuzzy prediction information in the recurrent process increases the convergence speed of the training.

### Strengthening memory layer

LSTM can learn long-term dependencies through deliberate design, and the critical component is the memory cell. To strengthen the long-term memory and alleviate the gradient dispersion problem of LSTM, the output needs to be determined by the current output and the cell state, thus the strengthening memory layer is proposed. In the strengthening memory layer, the current output and the cell state are combined to form a new unit. Then, the convolution Conv1d and tanh functions are used to extract more effective features to form the new memory cell. Finally, the output is generated by adding the current and new cell states, and it can be computed as follows:

17 $$\begin{aligned} ch_t=h_t+c_t \end{aligned}$$

18 $$\begin{aligned} s_t=\textrm{tanh}({ \mathrm Conv1d}([ch_t])) \end{aligned}$$

19 $$\begin{aligned} \hat{h}_t=ch_t+s_t \end{aligned}$$

Due to the addition of the new state, the latest information can be strengthened, and through the addition of new features, more information can be saved. The results of two kinds of feature information are combined in a summation way, which can strengthen the impact of the new state on the final result to a certain extent and make the results more comprehensive.

### Parameter segment sharing strategy

Parameter sharing is a necessary method for controlling the number of model parameters, which improves the efficiency of the model. Parameter sharing is a reduction of the parameters that the model has to learn, which make the model processing more efficient. However, this also results in coupled optimization among different candidates, making architectures less discriminative. Therefore, a strategy of parameter segment sharing towards better trade-off between processing efficiency and architecture discrimination is proposed for LSTM. Let the prediction length be *L*, and the number of shared parameters be *s*, the $k=L/s$ output layers are constructed to predict. Different output layers can capture temporal features from different time periods, which improves the architecture discrimination. Meanwhile, the output layer with *s* shared parameters guarantees the model processing efficiency. Finally, the output layer can be expressed in the form:

20 $$\begin{aligned} y_{t}=W_{yk}\cdot \hat{h}_t+b_{y} \end{aligned}$$

where $y_{t}$ is the forecast result, $W_{yk}$ is weight matrices, $\hat{h}_t$ is the output of the strengthen layer, and $b_y$ is bias.

### FLSTM model

FLSTM is based on the LSTM model and integrates the fuzzy system to leverage the advantages of both fuzzy logic and deep learning. FLSTM combines the fuzzy prediction fusion, the strengthening memory layer, and the parameter segment sharing strategy to enhance the accuracy and interpretability of LSTM for long-term time series prediction. First, the proposed fuzzy prediction model is utilized to obtain the fuzzy rule-based prediction value. This information will be fused into the input gate, forget gate, and output gate of LSTM. Then, the strengthening memory layer sums the hidden state and the cell state to form a new state, and extracts more effective features using convolution and tanh functions. Add the new state and the new state after feature extraction to generate the strengthening memory state. The parameter segment sharing strategy can be flexibly adjusted based on different datasets and various transformations of prediction cycles and lengths, improving the model’s ability to extract periodic features from time series data and effectively manages the increasing of network parameter. Algorithm 1 shows the details of the FLSTM model.

**Algorithm 1:**
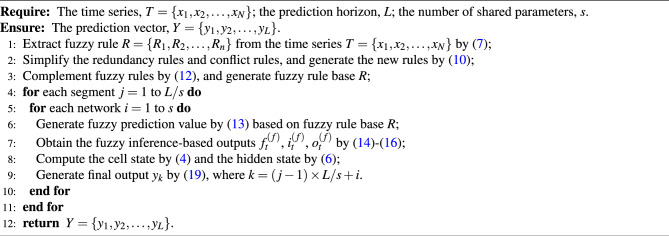
Fuzzy inference-based LSTM (FLSTM).

## Experimental study

To verify the prediction performance of FLSTM, a comparison with twenty-two prediction methods on seven collected real-world datasets is conducted. twenty-two time series prediction methods are selected for the comparative experiments, including three classical prediction method ARIMA^11^, SVR^35^, naive^36^, six deep learning-based prediction methods GRU^37^, DRNN^38^, LSTM^39^, Reformer^22^, LogSparse self-attention^23^, and Efficient attention^40^, seven LSTM-based fuzzy inference methods FD-LSTM^41^, FIS-LSTM^42^, SEIT2FNN^43^, RIT2NFS-WB^44^, MclT2FIS-UM^45^, MclT2TIS-US^45^, eIT2FNN-LSTM^46^, a LSTM-based fuzzy gaussian prediction method LFIGLSTM^30^, a fuzzy gaussian based fuzzy inference prediction method LFIGFIS^47^, a fuzzy prediction method FPFTS^48^, and a hybrid method MLP-Arima^8^, a nonlinear autoregressive neural network NAR^49^. Seven real-world datasets are the crucial indicators in the electric power deployment, air quality assessment, daily number of Covid-19 cases, monthly sunspot numbers, and daily maximum temperatures, i.e. Electricity Transformer Temperature (ETT)^50^, PM2.5^51^, daily Covid-19 cases^52^, monthly sunspot numbers^53^, daily maximum temperatures^54^, abalone age^51^, mile per gallon^51^. To evaluate the prediction effectiveness of the proposed method, the six performance indexes, MSE, MAE, RMSE, SMAPE, MAPE, and MASE are adopted^8, 40, 48^. For the sake of fairness, the selection of prediction length is consistent with the the original paper of the compared models for different datasets. The results of the compared models are derived from reports in the original paper.

### Experiment 1: Electricity Transformer Temperature time series

**Figure 4:**
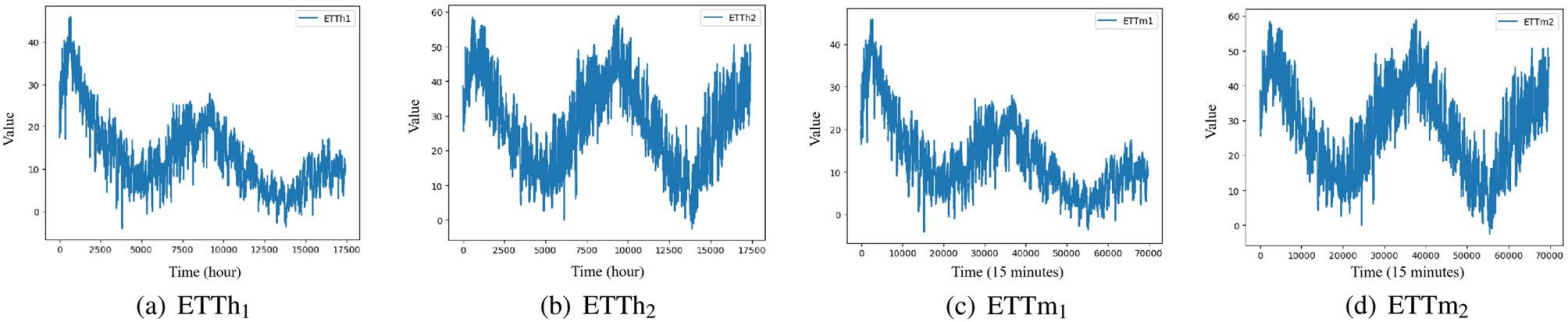
Illustration of Electricity Transformer Temperature (ETT) time series.

These time series are collected from Electricity Transformer Temperature (ETT)^50^ in Fig. 4, where $\textrm{ETTh}_{1}$ and $\textrm{ETTh}_{2}$ are created for 1-hour-level of 2 years data from two separated countries in China, and $\textrm{ETTm}_{1}$ and $\textrm{ETTm}_{2}$ are created for 15-minutes-level from the same datasets. Experimental parameters are set as follows: The update learning of parameters used Adam optimizer, the batch size is set to 32, the learning rate is set to 0.001, the training epoch is set to 100, the experiments times is set to 6, the dimension of the hidden layer is set to 64, the input and output channel are set to 64 for the Conv1d in the strengthening memory layer.

For $\textrm{ETTh}_{1}$ and $\textrm{ETTh}_{2}$ time series, the prediction lengths are set to 3, 6, 12, 18, 24, 36, 48, and 168 used for the experiment. For $\textrm{ETTm}_{1}$ and $\textrm{ETTm}_{2}$ time series, the prediction lengths are set to 4, 8, 12, 16, 24, 32, 48, 96, and 288. The prediction performance evaluation of ARIMA^11^, GRU^37^, DRNN^38^, LSTM^39^, FD-LSTM^41^, FIS^42^, Reformer^22^, LogTrans^23^, Efficient-att^40^, and the proposed method with different prediction lengths on the 4 time series are listed in Tables 1, 2, 3 and 4. The best results are highlighted in boldface and the winning-counts are listed in the last column.

From Tables 1, 2, we can see that FLSTM achieves better results than LSTM on MSE by decreasing 19.9% (at 48) and 29.0% (at 168) in average. This reveals that FLSTM significantly improves the performance of LSTM. In comparison with ARIMA, GRU, DRNN, Reformer, and LogTrans, FLSTM outperforms the prediction performances of these method across all datasets. FLSTM beats Efficient-att mostly in winning-counts, i.e. $14>3$ and $15>3$, and surpasses Efficient-att on longer length ($\ge 36$). From Tables 3, 4, we can see that FLSTM achieves better results than LSTM on MSE by decreasing 28.9% (at 96) and 32.4% (at 288) in average. This demonstrates that FLSTM significantly improves the performance of LSTM. Comparison with ARIMA, GRU, DRNN, LSTM, FD-LSTM, FIS and Reformer, FLSTM outperforms the prediction performances of these methods across all datasets. FLSTM beats LogTrans and Efficient-att mostly in winning-counts, i.e. $12>8$ and $12>1$ for $\textrm{ETTm}_{1}$, $12>4$ and $12>4$ for $\textrm{ETTm}_{2}$. The experiment shows that the success of FLSTM in enhancing the prediction performance in the long-term prediction problem.

**Table 1:** Time series forecasting results on $\textrm{ETTh}_1$ dataset.

Models	Metric	3	6	12	18	24	36	48	168	Count
ARIMA^11^	MSE	0.066	0.073	0.087	0.103	0.116	0.173	0.214	0.396	0
	MAE	0.174	0.211	0.234	0.243	0.254	0.383	0.413	0.504	
GRU^37^	MSE	0.006	0.015	0.027	0.036	0.044	0.164	0.197	0.213	0
	MAE	0.063	0.087	0.128	0.140	0.153	0.335	0.385	0.362	
DRNN^38^	MSE	0.006	0.016	0.025	0.034	0.043	0.161	0.197	0.207	0
	MAE	0.061	0.086	0.124	0.138	0.151	0.332	0.385	0.358	
LSTM^39^	MSE	0.006	0.015	0.025	0.034	0.041	0.163	0.195	0.236	0
	MAE	0.061	0.086	0.124	0.137	0.149	0.334	0.384	0.392	
FD-LSTM^41^	MSE	0.007	0.020	0.029	0.036	0.043	0.171	0.187	0.223	0
	MAE	0.065	0.094	0.131	0.144	0.154	0.341	0.378	0.386	
FIS^42^	MSE	0.005	0.015	0.024	0.036	0.041	0.151	0.186	0.217	0
	MAE	0.060	0.089	0.126	0.145	0.156	0.327	0.376	0.379	
Reformer^22^	MSE	0.011	0.027	0.041	0.067	0.081	0.237	0.263	1.522	0
	MAE	0.073	0.127	0.156	0.211	0.223	0.414	0.463	1.191	
LogTrans^23^	MSE	0.006	0.015	0.024	0.034	0.043	0.142	0.163	0.207	0
	MAE	0.062	0.087	0.123	0.139	0.156	0.312	0.365	0.375	
Efficient-att^40^	MSE	0.004	0.012	0.023	0.031	**0.037**	0.137	0.164	0.224	3
	MAE	0.056	**0.084**	0.121	0.132	**0.147**	0.307	0.365	0.384	
FLSTM(Ours)	MSE	**0.003**	**0.011**	**0.021**	**0.029**	0.038	**0.135**	**0.154**	**0.198**	14
	MAE	**0.052**	**0.084**	**0.117**	**0.127**	0.149	**0.303**	**0.347**	**0.362**

**Table 2:** Time series forecasting results on $\textrm{ETTh}_2$ datasets.

Models	Metric	3	6	12	18	24	36	48	168	Count
ARIMA^11^	MSE	2.674	3.454	3.641	3.617	3.517	3.347	3.136	2.800	0
	MAE	0.374	0.423	0.435	0.439	0.442	0.459	0.463	0.595	
GRU^37^	MSE	0.007	0.019	0.024	0.038	0.045	0.169	0.204	0.363	0
	MAE	0.067	0.094	0.125	0.143	0.156	0.332	0.387	0.492	
DRNN^38^	MSE	0.007	0.018	0.024	0.036	0.043	0.168	0.202	0.381	0
	MAE	0.064	0.092	0.124	0.140	0.156	0.330	0.386	0.501	
LSTM^39^	MSE	0.007	0.018	0.023	0.035	0.043	0.166	0.201	0.385	0
	MAE	0.065	0.091	0.124	0.140	0.156	0.330	0.386	0.514	
FD-LSTM^41^	MSE	0.007	0.019	0.026	0.034	0.043	0.171	0.183	0.337	0
	MAE	0.061	0.087	0.137	0.141	0.149	0.334	0.403	0.468	
FIS^42^	MSE	0.007	0.017	0.021	0.039	0.046	0.160	0.176	0.357	0
	MAE	0.063	0.084	0.119	0.149	0.155	0.326	0.395	0.470	
Reformer^22^	MSE	0.015	0.031	0.045	0.070	0.087	0.255	0.313	1.029	0
	MAE	0.087	0.135	0.163	0.223	0.234	0.453	0.482	0.879	
LogTrans^23^	MSE	0.007	0.017	0.023	0.036	0.045	0.160	0.168	0.246	0
	MAE	0.064	0.089	0.121	0.143	0.157	0.330	0.373	0.422	
Efficient-att^40^	MSE	**0.006**	**0.014**	0.022	0.033	0.041	0.158	0.167	0.263	3
	MAE	0.061	**0.076**	0.120	0.137	0.152	0.329	0.375	0.441	
FLSTM(Ours)	MSE	**0.006**	**0.014**	**0.020**	**0.032**	**0.039**	**0.154**	**0.163**	**0.243**	15
	MAE	**0.059**	0.079	**0.117**	**0.136**	**0.149**	**0.322**	**0.371**	**0.418**

**Table 3:** Time series forecasting results on $\textrm{ETTm}_1$ datasets.

Models	Metric	4	8	12	16	24	32	48	96	288	Count
ARIMA^11^	MSE	0.043	0.064	0.079	0.086	0.093	0.135	0.167	0.272	0.462	0
	MAE	0.182	0.203	0.214	0.219	0.221	0.264	0.303	0.399	0.558	
GRU^37^	MSE	0.004	0.006	0.006	0.012	0.021	0.094	0.109	0.304	0.536	0
	MAE	0.032	0.047	0.051	0.076	0.117	0.244	0.263	0.433	0.597	
DRNN^38^	MSE	0.004	0.005	0.006	0.011	0.020	0.092	0.107	0.301	0.557	0
	MAE	0.031	0.045	0.049	0.074	0.114	0.243	0.262	0.433	0.611	
LSTM^39^	MSE	0.004	0.005	0.041	0.011	0.057	0.092	0.107	0.287	0.524	0
	MAE	0.031	0.123	0.151	0.074	0.178	0.243	0.262	0.420	0.584	
FD-LSTM^41^	MSE	0.005	0.006	0.007	0.010	0.024	0.083	0.103	0.255	0.491	0
	MAE	0.034	0.043	0.054	0.072	0.116	0.232	0.264	0.431	0.561	
FIS^42^	MSE	0.005	0.006	0.008	0.011	0.021	0.078	0.096	0.241	0.452	0
	MAE	0.032	0.045	0.052	0.070	0.109	0.233	0.271	0.433	0.521	
Reformer^22^	MSE	0.020	0.037	0.056	0.081	0.095	0.114	0.163	0.920	1.108	0
	MAE	0.093	0.141	0.184	0.235	0.252	0.253	0.286	0.767	1.245	
LogTrans^23^	MSE	**0.003**	0.005	0.006	0.010	0.019	0.078	0.085	0.199	0.411	1
	MAE	0.031	0.047	0.051	0.072	0.111	0.234	0.254	0.386	0.572	
Efficient-att^40^	MSE	**0.003**	**0.004**	**0.005**	0.009	**0.017**	0.074	**0.083**	0.227	0.463	8
	MAE	0.029	0.041	**0.044**	0.068	**0.107**	0.251	**0.251**	0.413	0.593	
FLSTM(Ours)	MSE	**0.003**	**0.004**	0.005	**0.008**	0.018	**0.071**	0.090	**0.194**	**0.382**	12
	MAE	**0.028**	**0.039**	0.047	**0.064**	0.109	**0.225**	0.256	** 0.384**	**0.508**

**Table 4:** Time series forecasting results on $\textrm{ETTm}_2$ datasets.

Models	Metric	4	8	12	16	24	32	48	96	288	Count
ARIMA^11^	MSE	1.439	1.974	2.122	2.164	2.234	2.316	2.361	2.731	2.804	0
	MAE	0.378	0.423	0.434	0.438	0.441	0.453	0.463	0.571	0.714	
GRU^37^	MSE	0.004	0.006	0.011	0.015	0.024	0.091	0.115	0.281	0.464	0
	MAE	0.032	0.055	0.077	0.090	0.124	0.241	0.266	0.586	0.614	
DRNN^38^	MSE	0.004	0.006	0.011	0.016	0.023	0.088	0.114	0.296	0.621	0
	MAE	0.033	0.055	0.077	0.092	0.122	0.240	0.264	0.473	0.625	
LSTM^39^	MSE	0.004	0.006	0.011	0.015	0.023	0.087	0.112	0.271	0.673	0
	MAE	0.032	0.056	0.076	0.089	0.122	0.239	0.264	0.443	0.674	
FD-LSTM^41^	MSE	0.004	0.006	0.011	0.015	0.023	0.086	0.097	0.287	0.473	0
	MAE	0.033	0.057	0.087	0.091	0.119	0.231	0.249	0.433	0.615	
FIS^42^	MSE	0.004	0.006	0.010	0.014	0.024	0.078	0.087	0.264	0.563	0
	MAE	0.031	0.054	0.081	0.094	0.136	0.232	0.267	0.427	0.644	
Reformer^22^	MSE	0.023	0.041	0.062	0.086	0.102	0.134	0.185	0.874	0.956	0
	MAE	0.095	0.147	0.193	0.243	0.264	0.263	0.295	0.663	0.823	
LogTrans^23^	MSE	0.004	0.006	0.012	0.014	0.022	0.080	**0.086**	0.213	** 0.397**	4
	MAE	0.034	0.054	0.079	0.087	0.121	0.232	**0.243**	0.397	**0.551**	
Efficient-att^40^	MSE	0.004	**0.005**	0.010	0.013	0.020	0.077	**0.086**	0.224	0.436	4
	MAE	0.030	0.052	0.071	**0.082**	**0.116**	0.230	0.245	0.412	0.576	
FLSTM(Ours)	MSE	**0.003**	**0.005**	**0.009**	**0.012**	**0.019**	**0.074**	0.087	**0.203**	0.427	12
	MAE	**0.029**	**0.050**	**0.069**	0.113	0.166	**0.227**	0.245	**0.387**	0.574	

### Experiment 2: PM2.5 time series

**Figure 5:**
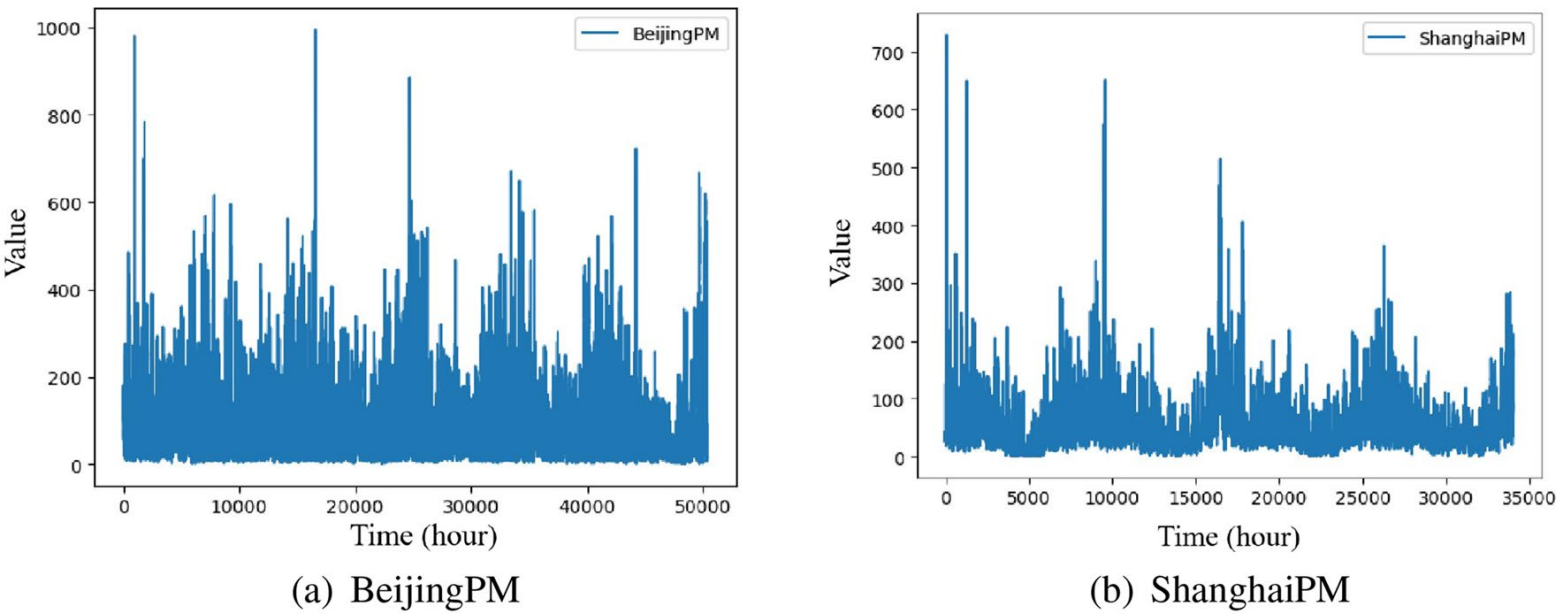
Illustration of PM2.5 time series.

Currently, research on PM2.5 data has generated great enthusiasm, and more and more deep learning based models have been proposed and applied to long-term PM2.5 generation^55, 56^. Therefore, we have increased our research on time series prediction of PM2.5 data. These time series are collected from PM2.5 data^51^ in Fig. 5, where BeijingPM and ShanghaiPM are the PM2.5 data of Bejing and Shanghai in China from 2010 to 2015, including 50387 and 51892 observations respectively. Experimental parameters are set as follows: The update learning of parameters used Adam optimizer, the batch size is set to 32, the learning rate is set to 0.001, the training epoch is set to 100, the experiments times is set to 6, the dimension of the hidden layer is set to 64, the input and output channel are set to 64 for the Conv1d in the strengthening memory layer. The prediction lengths are set to 200, 400, and 600 used for the experiment. Tables 5 and 6 summarize the evaluation results of ARIMA^11^, LSTM^39^, FPFTS^48^, and FLSTM with the three long-term prediction lengths. The best results are highlighted in boldface and the winning counts are listed in the last row.

Table 5 demonstrates that FLSTM outperforms other methods for the PM2.5 time series of Beijing in terms of all evaluation metrics, except that FPFTS has the smallest RMSE when the prediction length is 600. The proposed method surpasses FPFTS mostly in winning-counts, i.e. $5>1$. In comparison with LSTM, the proposed method has a RMSE decrease of 7.0% (at 200), 8.0% (at 400), and 8.1% (at 600). This demonstrates FLSTM acquires better prediction performance than LSTM. From Table 6, we can see that FLSTM for the two evaluation metrics with all prediction lengths outperforms ARIMA, LSTM, and WM for the PM2.5 time series of Shanghai. FLSTM surpasses FPFTS mostly in winning-counts, i.e. $4>2$. In comparison with LSTM, FLSTM has an RMSE decrease of 38.2% (at 200), 26.6% (at 400), and 16.7% (at 600). This demonstrates FLSTM acquires better prediction performance than LSTM. The experiment shows that the success of FLSTM in improving the prediction capacity for long-term prediction.

**Table 5:** Time series forecasting results on PM2.5 time series of Beijing.

Prediction length	Metric	ARIMA^11^	LSTM^39^	FPFTS^48^	FLSTM(Ours)
200	RMSE	269.425	189.279	185.049	**176.416**
	SMAPE	1.939	0.913	0.891	**0.871**
400	RMSE	239.308	193.850	186.940	**178.262**
	SMAPE	1.8951	1.039	0.979	**0.885**
600	RMSE	226.775	196.529	**171.223**	180.512
	SMAPE	1.947	1.036	0.934	**0.902**
	Count	0	0	1	5

**Table 6:** Time series forecasting results on PM2.5 time series of Shanghai.

Prediction length	Metric	ARIMA^11^	LSTM^39^	FPFTS^48^	FLSTM(Ours)
200	RMSE	24.207	6.401	4.221	**3.954**
	SMAPE	1.956	1.436	1.759	**1.142**
400	RMSE	23.382	7.107	5.776	**5.215**
	SMAPE	1.797	1.359	**1.100**	1.246
600	RMSE	24.781	7.986	7.337	**6.639**
	SMAPE	1.864	1.570	**0.939**	1.432
	Count	0	0	2	4

### Experiment 3: Daily number of Covid-19 cases time series

**Figure 6:**
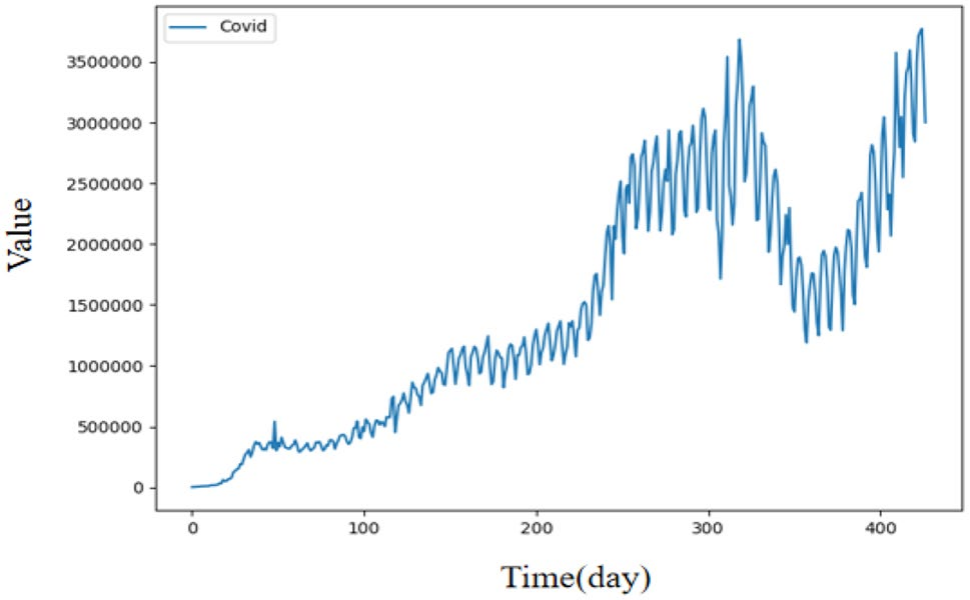
Illustration of the daily number of Covid-19 cases time series.

This time series is collected from the daily number of Covid-19 cases database owned by the organization Our World In Data (OWID)^52^, and it is built by the number of daily cases in the world until April 25th, 2021 in Fig. 6. Experimental parameters are set as follows: The update learning of parameters used Adam optimizer, the batch size is set to 32, the learning rate is set to 0.001, the training epoch is set to 100, the experiments times is set to 6, the dimension of the hidden layer is set to 64, the input and output channel are set to 64 for the Conv1d in the strengthening memory layer. The prediction lengths are set to 7, 14, and 28 used for the experiment same as in the literature^8^. The prediction performance evaluation of ARIMA(2,0,4)(0,1,2), MLP(14,5,1), MLP-Arima^8^, and FLSTM for short (7 days), medium (14 days), and long (28 days) prediction lengths are list in Table 7. The best results are highlighted in boldface and the winning counts are listed in the last row.

Table 7 demonstrates that FLSTM for MASE and SMAPE with all prediction lengths outperforms other methods for the daily number of Covid-19 cases time series. In comparison with the state-of-the-art method MLP-Arima^8^, FLSTM has a MASE decrease of 6.6% (at 7), 79.5% (at 14), and 19.1% (at 28). This demonstrates FLSTM acquires better prediction performance than MLP-Arima. From Table 7, we can see that FLSTM surpasses the comparative methods in all winning-counts. The experiment shows that the success of FLSTM in improving the prediction capacity for different prediction lengths.

**Table 7:** Time series forecasting results on the daily number of Covid-19 cases time series.

Prediction length	Metric	ARIMA(2,0,4)(0,1,2)	MLP(14,5,1)	MLP-Arima^8^	FLSTM(Ours)
7	MASE	0.544	0.358	0.290	**0.271**
	SMAPE	0.045	0.030	0.024	**0.021**
14	MASE	0.518	0.318	1.419	**0.291**
	SMAPE	0.044	0.027	0.110	**0.024**
28	MASE	1.264	1.026	0.587	**0.466**
	SMAPE	0.147	0.118	0.071	**0.054**
	Count	0	0	0	6

### Experiment 4: Monthly sunspot numbers time series

**Figure 7:**
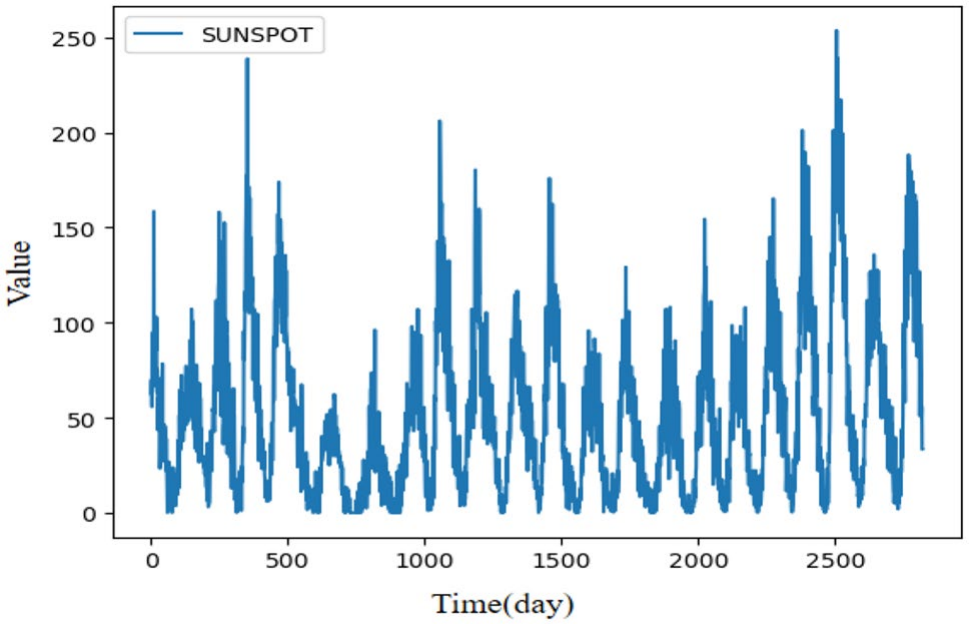
Illustration of the Zuerich monthly sunspot numbers time series.

This time series is collected from sunspot data in Fig. 7, where SUNSPOT^53^ is the sunspot data of Zuerich monthly sunspot numbers from 1749 to 1983, including 2819 observations respectively. Experimental parameters are set as follows: The update learning of parameters used Adam optimizer, the batch size is set to 32, the learning rate is set to 0.001, the training epoch is set to 100, the experiments times is set to 6, the dimension of the hidden layer is set to 64, the input and output channel are set to 64 for the Conv1d in the strengthening memory layer. The prediction lengths are set to 1, 55, 110, and 165 used for the experiment. Table 8 summarizes the evaluation results of LFIGLSTM^30^, LFIGFIS^47^, LSTM^39^, NAR^49^, ARIMA^11^, SVR^35^, naive^36^ and FLSTM with four prediction lengths. The best results are highlighted in boldface and the winning counts are listed in the last column.

Table 8 demonstrates that FLSTM for RMSE, MAPE and MAE with all prediction lengths outperforms other methods for the monthly sunspot numbers time series. In comparison with the state-of-the-art method LFIGLSTM, FLSTM has a RMSE decrease of 85.2% (at 1), 50.5% (at 55), 34.8% (at 110) and 27.2% (at 165). This demonstrates FLSTM acquires better prediction performance than LFIGLSTM. From Table 8, we can see that FLSTM surpasses the comparative methods in all winning-counts. The experiment shows that FLSTM has advantages over classical prediction models, deep learning prediction models, and hybrid prediction models in both short-term and long-term prediction tasks.

**Table 8:** Time series forecasting results on monthly sunspot numbers.

Model	Forecasting horizon:1			Forecasting horizon:55			Forecasting horizon:110			Forecasting horizon:165			Count
Metric	RMSE	MAPE	MAE	RMSE	MAPE	MAE	RMSE	MAPE	MAE	RMSE	MAPE	MAE	
LFIGLSTM^30^	14.612	13.682	14.612	20.696	21.509	16.954	18.969	55.791	15.434	32.307	47.209	24.528	0
LFIGFIS^47^	10.316	9.872	10.306	24.253	24.579	20.477	27.489	121.812	23.159	52.337	98.886	40.532	0
LSTM^39^	8.194	7.673	8.194	21.770	23.405	17.682	22.842	71.876	18.482	41.981	62.205	32.068	0
NAR^49^	20.250	18.961	24.250	32.297	52.553	27.575	50.728	272.470	44.506	57.887	194.773	48.234	0
ARIMA^11^	15.885	14.873	15.885	26.365	42.677	21.719	37.938	204.281	33.098	57.682	154.589	48.361	0
SVR^35^	17.322	16.219	17.322	35.650	59.204	31.000	54.617	293.199	48.120	56.345	207.345	49.468	0
Naive^36^	29.000	27.154	29.000	65.011	108.351	59.438	90.398	463.493	83.706	75.797	315.881	63.878	0
FLSTM	**2.167**	**2.809**	**2.167**	**10.358**	**12.644**	**11.587**	**12.364**	**15.372**	**13.625**	**23.504**	**28.248**	**21.648**	12

### Experiment 5: Daily maximum temperatures time series

**Figure 8:**
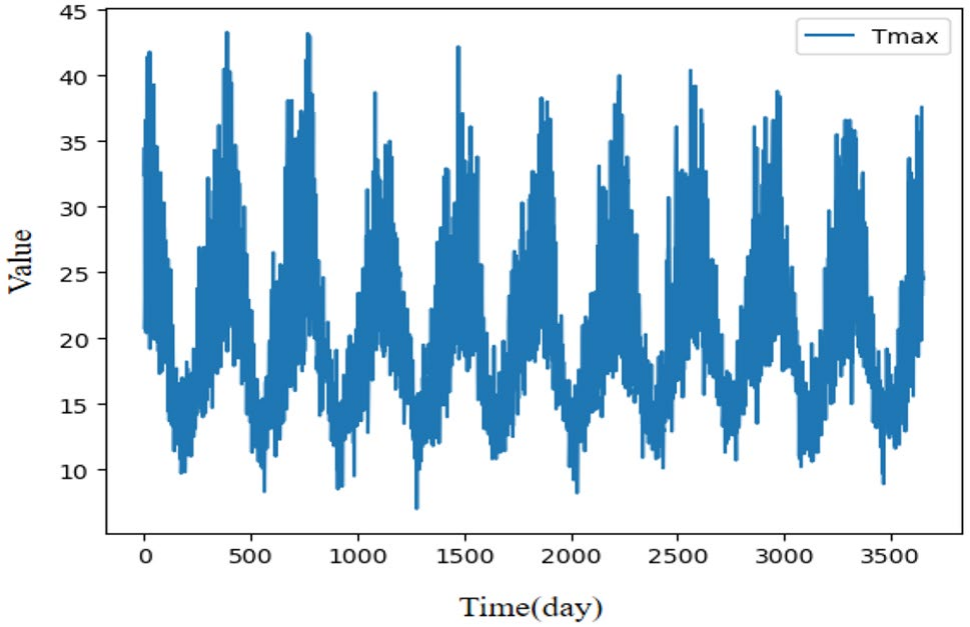
Illustration of the Melbournea daily maximum temperatures time series.

This time series is collected from temperature data in Fig. 8 where Tmax^54^ is the temperature data of daily maximum temperatures in Melbournea from 1981 to 1990, including 3649 observations respectively. Experimental parameters are set as follows: The update learning of parameters used Adam optimizer, the batch size is set to 32, the learning rate is set to 0.001, the training epoch is set to 100, the experiments times is set to 6, the dimension of the hidden layer is set to 64, the input and output channel are set to 64 for the Conv1d in the strengthening memory layer. The prediction lengths are set to 1, 178, 356, and 534 used for the experiment. Table 9 summarizes the evaluation results of LFIGLSTM^30^, LFIGFIS^47^, LSTM^39^, NAR^49^, ARIMA^11^, SVR^35^, naive^36^ and FLSTM with four prediction lengths. The best results are highlighted in boldface and the winning counts are listed in the last column.

Table 9 demonstrates that FLSTM outperforms other methods for the daily maximum temperatures in terms of all evaluation metrics, except that LFIGLSTM has the smallest MAE when the prediction length is 356 and 534. In comparison with the state-of-the-art method LFIGLSTM, FLSTM has a RMSE decrease of 18.9% (at 1), 13.8% (at 55), 15.4% (at 110) and 9.5% (at 165). The experiment shows that FLSTM has significant advantages over these prediction models in both short-term and long-term prediction tasks.

**Table 9:** Time series forecasting results on maximum temperatures.

Model	Forecasting horizon:1			Forecasting horizon:178			Forecasting horizon:356			Forecasting horizon:534			Count
Metric	RMSE	MAPE	MAE	RMSE	MAPE	MAE	RMSE	MAPE	MAE	RMSE	MAPE	MAE	
LFIGLSTM^30^	1.031	7.258	1.031	4.208	15.757	3.170	4.050	14.813	**3.069**	4.255	15.081	**3.139**	2
LFIGFIS^47^	1.894	15.785	1.894	4.399	20.045	3.678	4.205	17.020	3.353	4.356	17.991	3.483	0
LSTM^39^	1.508	10.616	1.508	6.595	20.651	4.650	7.320	24.701	5.514	7.356	25.342	5.515	0
NAR^49^	1.369	9.643	1.369	7.112	34.726	6.049	7.406	35.326	6.262	7.767	38.174	6.653	0
ARIMA^11^	1.290	9.083	1.290	5.593	16.709	3.721	6.075	20.216	4.407	6.018	21.583	4.486	0
SVR^35^	1.351	9.513	1.351	5.504	21.606	4.124	5.946	21.926	4.497	5.972	22.193	4.518	0
Naive^36^	2.300	16.197	2.300	9.251	32.986	7.253	10.146	36.283	8.263	9.942	35.351	7.992	0
FLSTM	**0.837**	**4.534**	**0.837**	**3.626**	**12.351**	**2.963**	**3.427**	**12.251**	3.164	**3.851**	**13.542**	3.267	10

### Experiment 6: Abalone age time series

**Figure 9:**
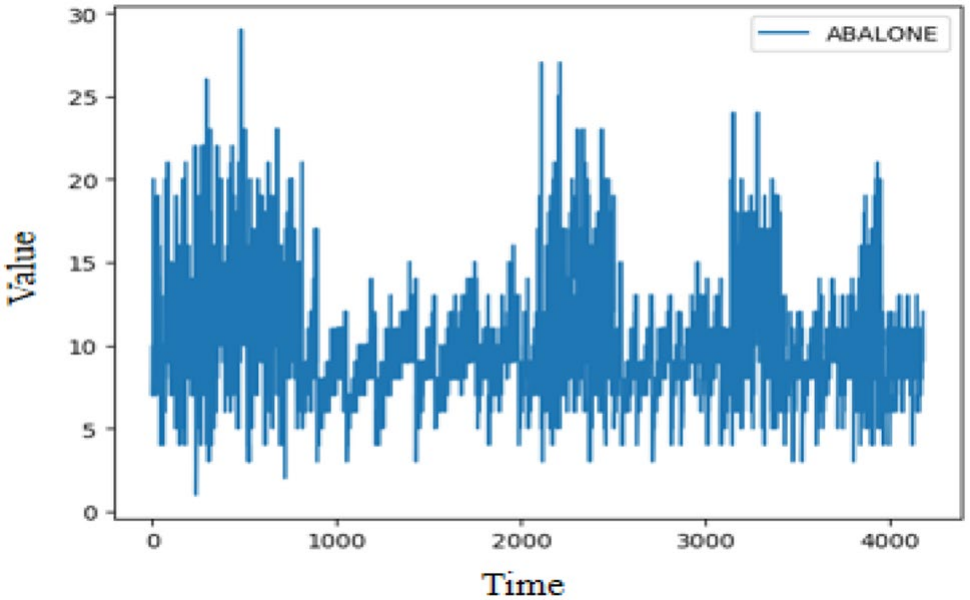
Illustration of abalone age time series.

Abalone age (ABALONE) time series^51^ is collected from the UCI machine learning repository as shown in Fig. 9, which includes 4177 observations. Experimental parameters are set as follows: The update learning of parameters used Adam optimizer, the learning rate is set to 0.001, the training epoch is set to 200, the experiment times is set to 6, the dimension of the hidden layer is set to 64, the input and output channel are set to 64 for the Conv1d in the strengthening memory layer. The prediction length is set to 835 used for the experiment. Table 10 summarizes the evaluation results of SEIT2FNN^43^, RIT2NFS-WB^44^, MclT2FIS-UM^45^, MclT2TIS-US^45^, eIT2FNN-LSTM^46^, and FLSTM. The best result is highlighted in boldface.

Table 10 demonstrates that FLSTM outperforms other methods for the abalone age prediction problem in terms of RMSE. In comparison with the state-of-the-art method eIT2FNN-LSTM, FLSTM has a RMSE decrease of 4.5%. The experiment shows that FLSTM has significant advantages over these prediction models.

**Table 10:** Time series forecasting results on abalone age prediction.

Algorithm	Training RMSE	Test RMSE
SEIT2FNN^43^	2.338	2.433
RIT2NFS-WB^44^	2.404	2.134
MclT2FIS-UM^45^	2.348	1.874
MclT2TIS-US^45^	2.335	1.838
eIT2FNN-LSTM^46^	1.190	1.533
FLSTM	**0.973**	**1.384**

### Experiment 7: Miles-Per-Gallon time series

**Figure 10:**
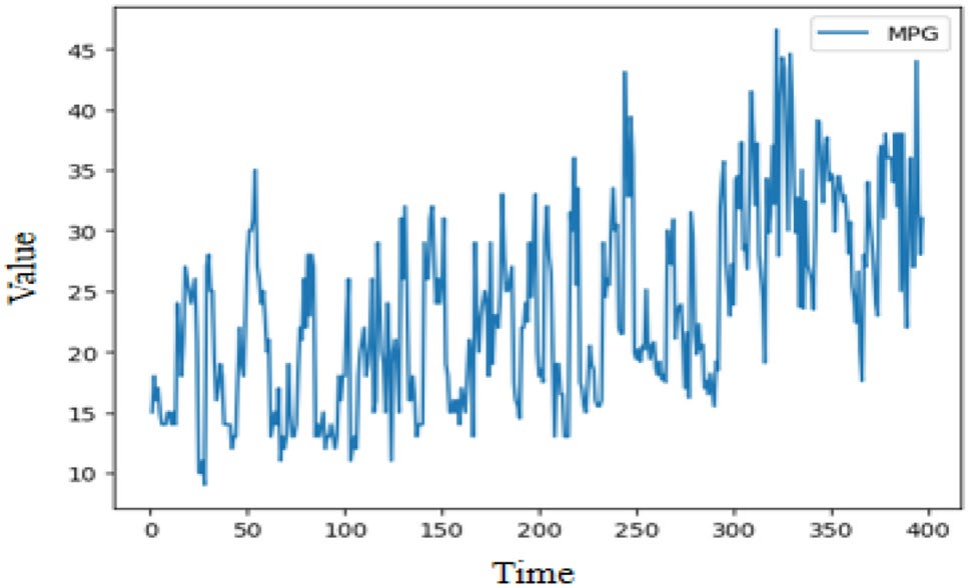
Illustration of Miles-Per-Gallon time series.

Miles-Per-Gallon (MPG)^51^ time series is collected from the UCI machine learning repository in Fig. 10 which includes 392 observations. Experimental parameters are set as follows: The update learning of parameters used Adam optimizer, the learning rate is set to 0.001, the training epoch is set to 200, the experiment times is set to 6, the dimension of the hidden layer is set to 64, the input and output channel are set to 64 for the Conv1d in the strengthening memory layer. The prediction length is set to 120 used for the experiment. Table 11 summarizes the evaluation results of SEIT2FNN^43^, RIT2NFS-WB^44^, MclT2FIS-UM^45^, MclT2TIS-US^45^, eIT2FNN-LSTM^46^, and FLSTM. The best result is highlighted in boldface.

Table 11 demonstrates that FLSTM outperforms other methods for the Miles-Per-Gallon prediction problem in terms of RMSE. In comparison with the state-of-the-art method eIT2FNN-LSTM, FLSTM has a RMSE decrease of 9.7%. The experiment shows that FLSTM has significant advantages over these prediction models.

**Table 11:** Time series forecasting results on Mile-Per-Gallon prediction.

Algorithm	Training RMSE	Test RMSE
SEIT2FNN^43^	2.7161	2.7895
RIT2NFS-WB^44^	2.3685	2.7807
MclT2FIS-UM^45^	2.6524	2.6486
MclT2TIS-US^45^	2.7358	2.6770
eIT2FNN-LSTM^46^	2.4731	2.5423
FLSTM	**1.8263**	**2.4186**

### Ablation study

To demonstrate the respective roles of different components in the proposed method, including the fuzzy prediction fusion (FPF), strengthening memory layer (SML), and parameter segment sharing (PSS) strategy, the ablation study on the $\textrm{ETTh}_1$ dataset is carried out. For a finer analysis, the experimental results vary with different combinations of LSTM, FPF, SML, and PSS are shown in Tables 12 and 13 for different prediction lengths.

Results presented in Tables 12 and 13 reveal that the proposed method FLSTM outperforms all other combinations of LSTM, FPF, SML, and PSS for short-term and long-term predictions in terms of MSE and MAE. The three combinations with different components all improve the accuracy of LSTM, which verifies the respective roles of FPF, SML, and PSS. Although the combinations with two different components also obtain the best results as the proposed method, such as LSTM+SML+PSS at short-term prediction lengths, the performances of the methods drop when one component is removed from the proposed method for all long-term prediction lengths. This is attributed to the fact that each component has a positive impact on improving prediction capacity. The proposed method gathers the benefits of the three improvement components and gets the best performance for all prediction lengths.

**Table 12:** Ablation study on the $\textrm{ETTh}_1$ dataset for short-term prediction.

Baseline	FPF	SML	PSS	Metric	3	12	24
LSTM				MSE	0.007	0.023	0.043
				MAE	0.065	0.124	0.155
LSTM	$\surd$			MSE	0.006	0.022	0.042
	$\surd$			MAE	0.061	0.121	0.152
LSTM		$\surd$		MSE	0.007	0.022	0.042
		$\surd$		MAE	0.063	0.124	0.155
LSTM			$\surd$	MSE	0.004	0.021	0.039
			$\surd$	MAE	0.056	0.117	0.148
LSTM	$\surd$	$\surd$		MSE	0.006	0.022	0.042
	$\surd$	$\surd$		MAE	0.061	0.121	0.152
LSTM	$\surd$		$\surd$	MSE	0.004	0.021	0.039
	$\surd$		$\surd$	MAE	0.058	0.118	0.149
LSTM		$\surd$	$\surd$	MSE	0.003	0.021	0.040
		$\surd$	$\surd$	MAE	0.052	0.119	0.149
LSTM	$\surd$	$\surd$	$\surd$	MSE	**0.003**	**0.021**	**0.038**
	$\surd$	$\surd$	$\surd$	MAE	**0.052**	**0.117**	**0.149**

**Table 13:** Ablation study on the $\textrm{ETTh}_1$ dataset for long-term prediction.

Baseline	FPF	SML	PSS	Metric	48	168	336	720
LSTM				MSE	0.190	0.385	0.558	0.640
				MAE	0.348	0.514	0.606	0.681
LSTM	$\surd$			MSE	0.178	0.314	0.463	0.571
	$\surd$			MAE	0.336	0.453	0.514	0.612
LSTM		$\surd$		MSE	0.183	0.305	0.427	0.482
		$\surd$		MAE	0.341	0.453	0.514	0.612
LSTM			$\surd$	MSE	0.172	0.292	0.416	0.527
			$\surd$	MAE	0.335	0.447	0.508	0.631
LSTM	$\surd$	$\surd$		MSE	0.176	0.273	0.386	0.476
	$\surd$	$\surd$		MAE	0.331	0.437	0.497	0.605
LSTM	$\surd$		$\surd$	MSE	0.168	0.312	0.434	0.506
	$\surd$		$\surd$	MAE	0.329	0.465	0.521	0.614
LSTM		$\surd$	$\surd$	MSE	0.168	0.254	0.364	0.464
		$\surd$	$\surd$	MAE	0.329	0.422	0.485	0.593
LSTM	$\surd$	$\surd$	$\surd$	MSE	**0.164**	**0.243**	**0.337**	**0.452**
	$\surd$	$\surd$	$\surd$	MAE	**0.328**	**0.418**	**0.476**	**0.582**

### Ethics declarations

There are not any experiments on humans and/or animals involved in this study.

## Conclusion

LSTM-based models yielded great success in the time series forecasting research field, but yet these methods have their main general drawbacks as accumulated error, diminishing temporal correlation, and laking interpretability. This research is undertaken to design a time series prediction model by integrating linear model Wang–Mendel fuzzy inference prediction method and LSTM network, which makes the model parameters more scientific and interpretable, and improves its performance in short-term time series prediction tasks. This study also aims to solve the problem of LSTM’s poor performance in long-term time series prediction tasks. We strengthened the long-term memory by using the strengthening memory layer, and balanced the processing efficiency and structural discrimination of the model by using the parameter segmentation sharing strategy, which solved the problem of LSTM’s poor performance in long-term time series prediction due to the gradient dispersion problem.

Seven publicly available time series are used to compare the prediction performance of the proposed method with eight methods, including three classical prediction method ARIMA, SVR, naive, six deep learning-based prediction methods GRU, DRNN, LSTM, Reformer, LogSparse self-attention, and Efficient attention, seven LSTM-Based Fuzzy inference methods FD-LSTM, FIS-LSTM, SEIT2FNN, RIT2NFS-WB, MclT2FIS-UM, MclT2TIS-US, eIT2FNN-LSTM, a LSTM-based fuzzy gaussian prediction method LFIGLSTM, a fuzzy gaussian based fuzzy inference prediction method LFIGFIS, a fuzzy prediction method FPFTS, and a hybrid method MLP-Arima, a nonlinear autoregressive neural network NAR. In comparison with the classical prediction method, FLSTM outperforms the prediction performances of the method across all datasets. In comparison with the hybrid method, FLSTM acquires better prediction performance for all prediction lengths. In comparison with deep learning-based prediction methods, FLSTM beats these methods in winning-counts. In comparison with the fuzzy prediction method, FLSTM outperforms the prediction performances of the method in terms of winning-count. The experiments show that the success of FLSTM in improving the prediction capacity for long-term prediction. FLSTM has disadvantages in computational complexity. FLSTM can only predict one step at a time, thus the time cost becomes larger as the prediction length increase. The fixed fuzzy rule generation mechanism also limits the flexibility of prediction. Of course, these also provide ideas for future research.

Future research will include the following: (1) support multi-step prediction at a time; (2) provide fuzzy reasoning with different cycle lengths; (3) extend LSTM network to more complex data; (4) apply the proposed method to other appealing directions.

## Data Availability

The datasets used in this study are publicly available.

## References

[1] Liu G, Xiao F, Lin CT (2020). A fuzzy interval time-series energy and financial forecasting model using network-based multiple time-frequency spaces and the induced-ordered weighted averaging aggregation operation. IEEE Trans. Fuzzy Syst..

[2] Bala R, Singh RP (2022). A dual-stage advanced deep learning algorithm for long-term and long-sequence prediction for multivariate financial time series. Appl. Soft Comput..

[3] Gao X, Cao Z, Li S (2019). Taxonomy and evaluation for microblog popularity prediction. ACM Trans. Knowl. Discov. Data (TKDD).

[4] Cao, Q., Shen, H. & Gao, J. Popularity prediction on social platforms with coupled graph neural networks. In *Proceedings of the 13th International Conference on Web Search and Data Mining*, 70–78 (2020).

[5] Chen X, Lan X, Wan J (2021). Evolutionary prediction of nonstationary event popularity dynamics of Weibo social network using time-series characteristics. Discret. Dyn. Nat. Soc..

[6] Sharma RR, Kumar M, Maheshwari S (2020). EVDHM-ARIMA-based time series forecasting model and its application for COVID-19 cases. IEEE Trans. Instrum. Meas..

[7] Shen F, Liu J, Wu K (2020). Multivariate time series forecasting based on elastic net and high-order fuzzy cognitive maps: A case study on human action prediction through EEG signals. IEEE Trans. Fuzzy Syst..

[8] de Araújo Morais LR, da Silva Gomes GS (2022). Forecasting daily Covid-19 cases in the world with a hybrid ARIMA and neural network model. Appl. Soft Comput..

[9] Dudek G, Pełka P, Smyl S (2021). A hybrid residual dilated LSTM and exponential smoothing model for midterm electric load forecasting. IEEE Trans. Neural Netw. Learn. Syst..

[10] Soda P, Sicilia R, Acciai L (2019). Grasping inter-attribute and temporal variability in multivariate time series. IEEE Trans. Big Data.

[11] Ariyo, A. A., Adewumi, A. O. & Ayo, C. K. Stock price prediction using the ARIMA model. In *2014 UKSim-AMSS 16th International Conference on Computer Modelling and Simulation*, 106–112 (2014).

[12] Panigrahi S, Behera HS (2017). A hybrid ETS-ANN model for time series forecasting. Eng. Appl. Artif. Intell..

[13] Geng X, Li H, Yao Z (2022). Potential of ANN for prolonging remote sensing-based soil moisture products for long-term time series analysis. IEEE Geosci. Remote Sens. Lett..

[14] Elman JL (1990). Finding structure in time. Cogn. Sci..

[15] Canizo M, Triguero I, Conde A (2019). Multi-head CNN-RNN for multi-time series anomaly detection: An industrial case study. Neurocomputing.

[16] Ni Q, Cao X (2022). MBGAN: An improved generative adversarial network with multi-head self-attention and bidirectional RNN for time series imputation. Eng. Appl. Artif. Intell..

[17] Hu, M., Jiang, K. & Nie, Z. You only align once: Bidirectional interaction for spatial-temporal video super-resolution. In *Proceedings of the 30th ACM International Conference on Multimedia*, 847–855 (2022).

[18] Ma C, Dai G, Zhou J (2021). Short-term traffic flow prediction for urban road sections based on time series analysis and LSTM\_BILSTM method. IEEE Trans. Intell. Transp. Syst..

[19] Bandara K, Bergmeir C, Hewamalage H (2020). LSTM-MSNet: Leveraging forecasts on sets of related time series with multiple seasonal patterns. IEEE Trans. Neural Netw. Learn. Syst..

[20] Vaswani A, Shazeer N, Parmar N (2017). Attention is all you need. Adv. Neural. Inf. Process. Syst..

[21] Xiao Y, Yuan Q, He J (2022). Space-time super-resolution for satellite video: A joint framework based on multi-scale spatial-temporal transformer. Int. J. Appl. Earth Obs. Geoinf..

[22] Kitaev, N., Kaiser, Ł. & Levskaya, A. Reformer: The efficient transformer. arXiv preprint arXiv:2001.04451 (2020).

[23] Li S, Jin X, Xuan Y (2019). Enhancing the locality and breaking the memory bottleneck of transformer on time series forecasting. Adv. Neural. Inf. Process. Syst..

[24] Zhou, H., Zhang, S. & Peng, J. Informer: Beyond efficient transformer for long sequence time-series forecasting. In *Proceedings of the AAAI Conference on Artificial Intelligence* **35**, 11106–11115 (2021).

[25] Garibaldi JM (2019). The need for fuzzy AI. IEEE/CAA J. Autom. Sin..

[26] Yeganejou M, Dick S, Miller J (2019). Interpretable deep convolutional fuzzy classifier. IEEE Trans. Fuzzy Syst..

[27] Zhang S, Sun Z, Wang M (2019). Deep fuzzy echo state networks for machinery fault diagnosis. IEEE Trans. Fuzzy Syst..

[28] Zhang Z, Yan Z (2019). An adaptive fuzzy recurrent neural network for solving the nonrepetitive motion problem of redundant robot manipulators. IEEE Trans. Fuzzy Syst..

[29] Li R, Hu Y, Liang Q (2020). T2F-LSTM method for long-term traffic volume prediction. IEEE Trans. Fuzzy Syst..

[30] Tang Y, Yu F, Pedrycz W (2021). Building trend fuzzy granulation-based LSTM recurrent neural network for long-term time-series forecasting. IEEE Trans. Fuzzy Syst..

[31] Wang LX (2003). The WM method completed: A flexible fuzzy system approach to data mining. IEEE Trans. Fuzzy Syst..

[32] Wang LX, Mendel JM (1992). Generating fuzzy rules by learning from examples. IEEE Trans. Syst. Man Cybern..

[33] Gou J, Hou F, Chen W (2015). Improving Wang-Mendel method performance in fuzzy rules generation using the fuzzy C-means clustering algorithm. Neurocomputing.

[34] Zhai Y, Lv Z, Zhao J (2021). Data-driven inference modeling based on an on-line Wang–Mendel fuzzy approach. Inf. Sci..

[35] Cortes C, Vapnik V (1995). Support vector machine. Mach. Learn..

[36] Webb GI, Keogh E, Miikkulainen R (2010). Naïve bayes. Encycl. Mach. Learn..

[37] Cho, K., Van Merriënboer, B. & Bahdanau, D. On the properties of neural machine translation: Encoder-Decoder approaches. arXiv preprint arXiv:1409.1259 (2014).

[38] Graves, A., Mohamed, A. R. & Hinton, G. Speech recognition with deep recurrent neural networks. In *2013 IEEE International Conference on Acoustics, Speech and Signal Processing*, 6645–6649 (2013).

[39] Bahdanau, D., Cho, K. & Bengio, Y. Neural machine translation by jointly learning to align and translate. arXiv preprint arXiv:1409.0473 (2014).

[40] Shen, Z., Zhang, M. & Zhao, H. Efficient attention: Attention with linear complexities. In *Proceedings of the IEEE/CVF Winter Conference on Applications of Computer Vision*, 3531–3539 (2021).

[41] Langeroudi MK, Yamaghani MR, Khodaparast S (2022). FD-LSTM: A fuzzy LSTM model for chaotic time-series prediction. IEEE Intell. Syst..

[42] Suppiah R, Kim N, Sharma A (2022). Fuzzy inference system (FIS)-long short-term memory (LSTM) network for electromyography (EMG) signal analysis. Biomed. Phys. Eng. Express.

[43] Juang C, Tsao Y (2008). A self-evolving interval type-2 fuzzy neural network with online structure and parameter learning. IEEE Trans. Fuzzy Syst..

[44] Juang C, Juang K (2012). Reduced interval type-2 neural fuzzy system using weighted bound-set boundary operation for computation speedup and chip implementation. IEEE Trans. Fuzzy Syst..

[45] Das AK, Subramanian K, Sundaram S (2015). An evolving interval type-2 neurofuzzy inference system and its metacognitive sequential learning algorithm. IEEE Trans. Fuzzy Syst..

[46] Wang H, Luo C, Wang X (2019). Synchronization and identification of nonlinear systems by using a novel self-evolving interval type-2 fuzzy LSTM-neural network. Eng. Appl. Artif. Intell..

[47] Yang X, Yu F, Pedrycz W (2017). Long-term forecasting of time series based on linear fuzzy information granules and fuzzy inference system. Int. J. Approx. Reason..

[48] Wang W, Liu W, Chen H (2022). Time series forecasting via fuzzy-probabilistic approach with evolving clustering-based granulation. IEEE Trans. Fuzzy Syst..

[49] Padilla, C., Hashemi, R. & Mahmood, N. H. A nonlinear autoregressive neural network for interference prediction and resource allocation in URLLC scenarios. In *2021 International Conference on Information and Communication Technology Convergence (ICTC)*, 184–189 (2021).

[50] ETT dataset. https://github.com/zhouhaoyi/ETDataset .

[51] UCI Machine Repository: Data Sets. http://archive.ics.uci.edu/ml/datasets.php.

[52] Coronavirus pandemic (covid-19). https://ourworldindata.org/coronavirus .

[53] Zurich monthly sunspot number. https://github.com/PacktPublishing/Practical-Time-Series-Analysis .

[54] Melbournea daily max temperatures. https://github.com/jbrownlee/Datasets .

[55] Alexeeff, S. E., Liao, N. S. & Liu, X. Long-term pm2.5 exposure and risks of ischemic heart disease and stroke events: review and meta-analysis. *J. Am. Heart Assoc.* **10**, e016890 (2021). 10.1161/JAHA.120.016890 PMC7955467 33381983

[56] Xiao Y, Wang Y, Yuan Q (2022). Generating a long-term (2003–2020) hourly 0.25 global PM2.5 dataset via spatiotemporal downscaling of CAMS with deep learning (DeepCAMS). Sci. Total Environ..

